# Rejecting an irrelevant singleton in the absence of a competing target

**DOI:** 10.3758/s13414-025-03069-8

**Published:** 2025-04-14

**Authors:** Luca Betteto, Matteo De Tommaso, Massimo Turatto

**Affiliations:** 1 Cimec, Center for Mind/Brain Sciences, University of Trento, Corso Bettini, 31, 38068 Rovereto, Italy; 2 Department of Psychology, University of Milano Bicocca, Milan, Italy

**Keywords:** Attentional capture, Visual search

## Abstract

Theories of visual distraction handling commonly propose that mechanisms of distractor rejection are engaged because irrelevant, salient objects tend to dominate the attentional competition with the target. Consequently, the resulting misallocation of attention is thought to trigger distractor suppression, ultimately reducing unwanted attentional capture. Using a modified version of the classic additional-singleton paradigm based on four consecutive displays in each trial, where the target and the distractor competed for attention only in the last one, we demonstrated that the attentional capture elicited by a color singleton was strongly attenuated when the singleton repeatedly appeared within the same trial, even in the absence of a competing target. Importantly, this capture attenuation was not associated with target impairment when the target appeared at the singleton location, suggesting that the within-trial rejection was likely controlled by an expectation-based mechanism rather than a suppressive one. Our findings point to the existence of distinct within-trial and across-trials rejection mechanisms, potentially operating on different timescales and involving suppressive and nonsuppressive processes.

## Introduction

How we learn to resist visual distraction is a widely debated topic in the attention literature. In particular, it has been shown that the brain can capitalize on the history of stimulation to filter out salient irrelevant distractors (Chelazzi et al., 2019; Geng et al., 2019; Lin et al., 2021; Sauter et al., 2021).

For the most part, these results have emerged from experiments adopting the additional-singleton paradigm (Theeuwes, 1992), which has become a sort of gold-standard approach to study attentional capture by feature-singleton distractors. Observers are typically asked to search for a shape singleton target, while on some trials a salient color singleton, serving as distractor, is presented in the display. Due to its higher salience, the color singleton tends to capture attention, as attested by the longer response times (RTs) on distractor-present compared with distractor-absent trials. However, the brain can progressively learn to suppress the distractor to limit the unwanted capture, and multiple lines of evidence indicate that distractor rejection is mainly experience based (e.g., Allenmark et al., 2022; Luck et al., 2021). The shared view is that distractor rejection would be implemented because at some level the brain implicitly realizes that the salient but irrelevant element tends to win the attentional competition with the target, thus producing an “erroneous” attentional selection. The mistake, where the distractor is selected instead of the target, serves as information to initiate a suppressive activity directed at the distractor, thus reducing its capacity to attract attention in the future.

This idea is clearly postulated in a recent review on the mechanisms of attentional capture and distraction handling: “The idea is that attention is captured (even for the briefest moment) by the salient singleton, and if it is not the target, it isimmediately suppressed” (see Theeuwes’s position in Luck et al., 2021, p. 14). Since in the additional-singleton paradigm the salient distractor is always presented with the target, it seems reasonable to assume that distractor suppression is triggered by a misallocation of attention with respect to the target element. Along the same line, Allenmark et al. (2022), who also used a variant of the classic additional-singleton paradigm, pointed out that “distractor-location probability learning is an active (though not necessarily explicit) process, that is: a process involving having to identify a distractor that captured attention as an erroneously selected item and to reject it (i.e., suppress its priority signal) to disengage attention and reorient it to the target item” (p. 1269). The same idea is also at the core of other similar proposals, like the *rapid disengagement hypothesis* (Belopolsky et al., 2010) or the *search and destroy hypothesis* (Moher & Egeth, 2012).

Despite this shared view, arising from the additional-singleton paradigm where the distractor, when present, is concurrent with the target, evidence exists indicating that learning to ignore irrelevant salient stimuli could also take place in the absence of a to-be-attended target element (Duncan & Theeuwes, 2020; Turatto et al., 2018a, 2018b; Won & Geng, 2020). Particularly, in the study of Turatto et al., (2018a, 2018b) and of Won and Geng (2020) the irrelevant potentially distracting stimuli were presented without any competing target element, so any attenuation of their capture capacity cannot be explained by the fact that they caused an erroneous attentional selection with respect to a competing target. While this seems to question the idea that an initial attentional erroneous selection in a situation of target–distractor competition is what drives the filtering mechanisms, these previous studies used heterogenous paradigms, often quite different from one another, or from the additional-singleton paradigm, where instead target and distractor directly compete for selection.

Hence, here we sought to address whether suppressing a salient element requires the occurrence of an erroneous attentional selection defined by the presence of a concurrent target element in the search display. To this aim, we used a variation of the classic additional-singleton task, in which the singleton distractor, on some trials, was presented also without a competing target. Specifically, each trial consisted of four consecutive discrete visual-search displays, with the target appearing only in the last display; however, a salient color-singleton stimulus, serving as a distractor when concomitant with the target, could appear either only in the last display, or also in the preceding three displays. The fact that on some trials an attentional grabbing stimulus was presented before the target is not per se a new experimental manipulation. There are at least two main paradigms in the literature in which a salient nontarget element is presented before the target. One is the well-known spatial-cueing paradigm devised by Posner and colleagues (e.g., Posner & Cohen, 1984), and the other one is a combination of the spatial cueing paradigm with a visual-search task, as in the paradigm used by Folk and colleagues to study attentional control (e.g., Folk et al., 1992). However, previous studies based on these paradigms did not address whether being rapidly exposed to an irrelevant salient singleton before the target changes its attention-grabbing power when it appears a few moments later in competition with the target. In other words, are the distractor rejection mechanisms engaged because the singleton wins the attentional competition with the concomitant target, or simply because it triggers an “irrelevant” attentional capture, as suggested by previous studies showing capture attenuation under passive viewing (e.g., Turatto et al., 2018a, 2018b)?

The paradigm characterizing the present set of experiments consists of four consecutive displays shown in rapid succession within the same trial, during which, depending on the concomitant or separate presence of the target and the distractor singleton, three conditions were possible. In the *absent* condition, the irrelevant singleton never appeared across the four displays, and the only singleton, appearing in the last display, was the target; in the *standard* condition, the first three displays no singleton was shown, whereas a color singleton appeared concomitantly with the target singleton in the fourth and last display, as in the classic additional-singleton paradigm; in the *repeated* condition, the color singleton started to appear from the first display, making it the only salient element in the first three displays, while the target was shown only in the last display.

Because the salient irrelevant color singleton was repeatedly presented not only across trials but also within the same trial, this allowed us also to investigate whether distractor rejection mechanisms might operate on different timescales—namely, across trials, as typically studied, and within the same trial (Pascucci & Turatto, 2015).

## Experiment 1

The main goal of the present experiment was to test whether the mere repeated presentation of a salient albeit irrelevant stimulus appearing at a fixed location, in the absence of any competing target, is sufficient to engage a filtering mechanism to attenuate its attentional grabbing power. Trials in which the singleton was iteratively presented four times in the same location defined the *repeated* condition, whereas trials in which the singleton appeared only in the fourth and last display (along with the target) defined the *standard* condition. If an erroneous allocation of attention with respect to the target location is a necessary condition to trigger distractor rejection mechanisms, then we expected to observe no significant effect of presenting the singleton in the first three displays—namely, there should be no difference in terms of capture (i.e., lengthening of RTs compared with the absent condition) between the standard and the repeated condition, because any rejection will be time-locked with the last display where the target is present. Indeed, in the absence of a target in the display, which defines the correct location where to direct attention, any other shifts attention cannot be strictly defined as erroneous. By contrast, if rejection can be initiated by the mere presence of a salient but irrelevant element in the scene, even when no competing target is concomitantly present, then we expected RTs to be significantly reduced in the repeated condition as compared with the standard one.

### Method

#### Participants

In all experiments, participants were recruited online via the Prolific platform (Prolific Academic Ltd, Oxford, UK). Participants had to be between 18 and 40 years old, fluent English speakers, to have normal or corrected-to-normal vision, and to run the experiment on a desktop computer. In the literature, there have been studies regarding the effect of distractor-location probability on distractor suppression that reported large effect sizes (e.g., Wang & Theeuwes, 2018). However, given that to the best of our knowledge, no prior studies employed our experimental manipulation, we were conservative and hence hypothesized a medium effect size. An a priori power analysis was conducted using G*Power 3.1.9.7 (Faul et al., 2007) to determine the minimum sample size required to test the study hypothesis. The results indicated that the required sample size to achieve 80% power for detecting a medium effect, at a significance criterion of $\alpha$ = 0.05, was *N* = 34 for a two-tailed *t* test. Eight participants were excluded for failing to reach the overall accuracy level of 75% of correct responses and were replaced by new participants to achieve the final sample size of 34 (six women; 28 men; mean age = 29.9 years). Participants were informed about the aim of the experiment, data handling, and were given the task instructions. They gave their consent by pressing a key to continue after the first explicative display, and they were paid 6 GBP for their participation. All the experiments of the present study were carried out in accordance with the Declaration of Helsinki, and with the approval of the local institutional ethics committee (Comitato Etico per la Sperimentazione con l’Essere Umano, Università degli Studi di Trento, Italy).

#### Stimuli and procedure

The experiment was programmed using the PsychoPy3 Version 2022.2.5 software (Peirce et al., 2019), and run online using the Pavlovia web hosting platform (Open Science Tools Limited, Nottingham, UK). Stimuli were defined in “height” units, allowing consistent resizing with different screens. Measures are reported in degrees of visual angle considering a 1,920 × 1,080 monitor and a viewing distance of 60 cm. Circles diameter was 2.53°, while diamonds were 2.05° × 2.05° units for both width and height. They were placed on the circumference of an imaginary circle, centered on the center of the screen, with a radius of 12.65°.

**Fig. 1:**
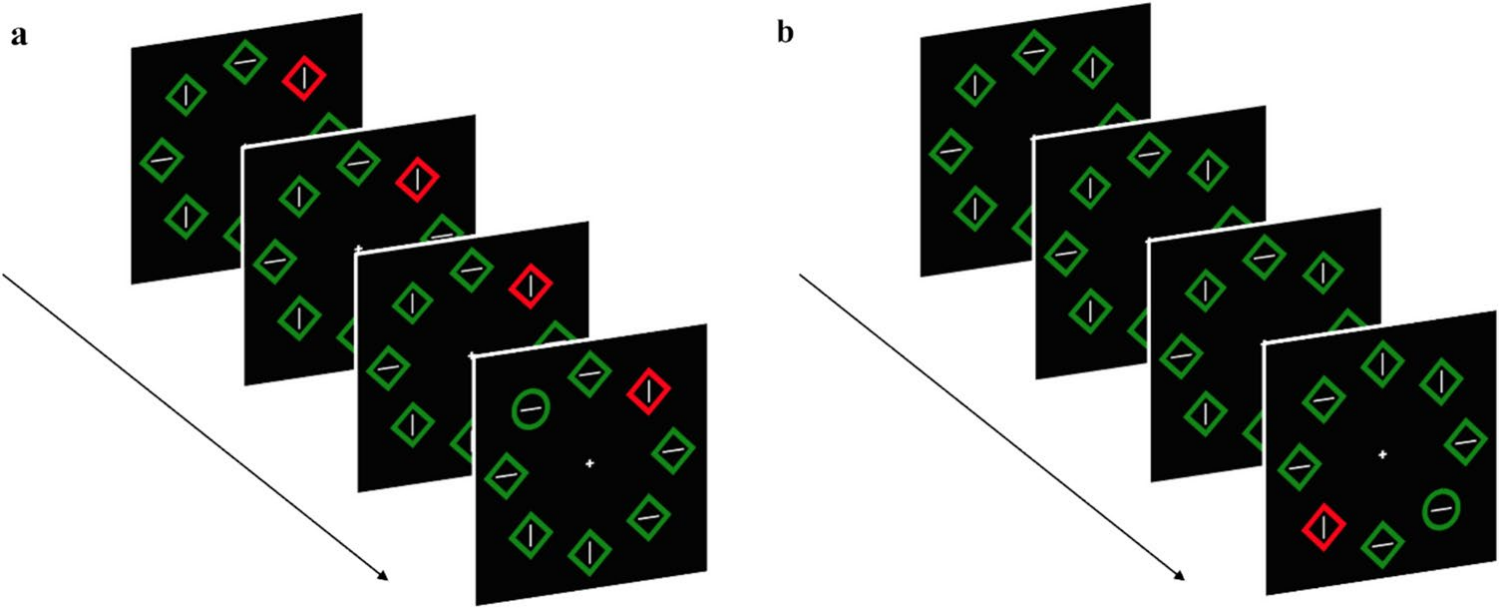
Schematic examples of a trial for the two distractor present conditions. Blanks between each display were omitted in the examples (see main text). In all conditions, the shape singleton target appeared only in the last display. In the *repeated* condition (**a**), one of the stimuli was a color singleton (it could be red among greens or vice versa) appearing in the same position in all four displays. In the *standard* condition (**b**), no singleton was presented in the first three displays, but it appeared only in the last display. The locations of the repeated and the standard conditions were fixed across trials. The *distractor*-*absent* condition (not shown in the figure) was as the standard condition, but no color singleton was presented in the last display. (Color figure online)

Each trial consisted of four consecutive displays, each presenting the following events (see Fig. 1): a white fixation cross (0.47° × 0.47°) was shown against a black background in the center of the screen for 500 ms; then eight geometrical figures appeared around the fixation point. They could be circles (2.53° diameter, 0.3° thickness), or outlined diamonds (2.05° × 2.05°, 0.3° thickness), with each figure embedding either a vertical or horizonal white line (1.5° length, 0.1° thickness). The figures lasted 300 ms in the first three displays, whereas in the last display they remained visible until a response was provided, or after 2,000 ms had elapsed. A blank interval of 500 ms separated each display. In the first three displays, all figures had the same shape and color (either red or green), except for the color singleton when present, and remained constant across the displays. In the fourth and last display one of the figures was a shape singleton, serving as the target (see Fig. 1b and c). The positions in which the singleton appeared, in the standard and repeated conditions, were fixed across trials for each participant, and were located on the opposing diagonals of the array of figures. These distractor positions were counterbalanced across participants.

Participants reported as fast and accurately as possible the orientation of the line inside the target element by pressing the right-arrow key on the keyboard if the line was horizontal, and the down-arrow key if the line was vertical. If a wrong answer was given, or the time to respond was exceeded, the words “Oops, wrong!” or “Try to be faster” appeared on the screen for 500 ms, while for correct responses a blank was shown. A blank 800-ms intertrial interval followed the participant response before the next trial began. Instructions were given to keep the gaze on the fixation cross throughout the experiment.

The experiment started with nine practice trials (three for each condition). The total number of trials was 300, split into two blocks of 150 each, separated by a 3-min pause. Completion time was approximately 40 min. The distractor-present trials were 60% of the total trials (180 out of 300) and were split equally into the standard and the repeated conditions (90 trials each). In each block, trials of each condition were randomly intermixed.

### Results and discussion

**Fig. 2:**
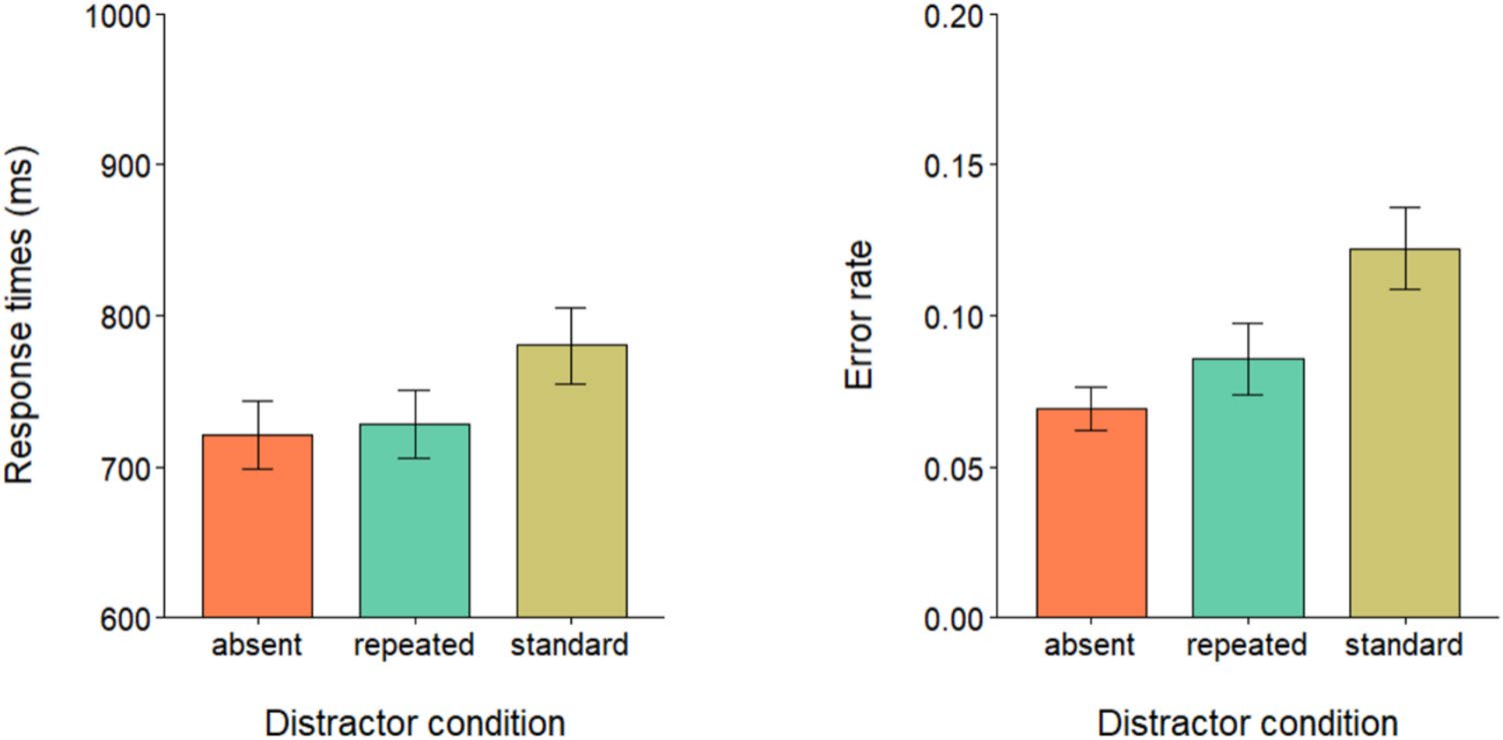
Mean RTs (left panel) and error rate proportions (right panel) in the three distractor conditions. Error bars denote the standard error of the mean

Data preprocessing and analyses were performed in RStudio (R Core Team, 2024). Response times (RTs) outliers (< 1%) were identified as RTs below 200 ms or above 2,000 ms and removed from further analysis. A repeated-measures analysis of variance (ANOVA) was performed on correct trials RTs (mean accuracy = 90.99%) to evaluate the effect of the three levels of the distractor condition: absent, standard, and repeated. Mauchly’s test indicated that the assumption of sphericity was violated (*W* = 0.69, *p* < 0.05). Degrees of freedom were corrected using Greenhouse–Geisser estimates of sphericity. The results showed a main effect of distractor condition, *F*(1.53, 50,66) = 51.20, *p* < 0.001, ${\eta }_{p}^{2}$ = 0.6. Post hoc comparisons with Bonferroni correction showed that participants in the standard condition (*M* = 780 ms; *SD* = 145 ms) were significantly slower than both in the repeated condition (*M* = 728 ms; *SD* = 129 ms), *t*(33) =  − 7.21, *p* < 0.001, *d* = − 1.24 and in the distractor-absent condition (*M* = 721 ms; *SD* = 130 ms), *t*(33) =  − 8.23, *p* < 0.001, *d* = − 1.41. No significant difference was found instead between the repeated condition and the distractor-absent condition *t*(33) =  − 1.61, *p* = 0.35 (see Fig. 2). The error rates were entered into a repeated-measures ANOVA, which showed a main effect of distractor condition, *F*(2, 66) = 16.64, *p* < 0.001, ${\eta }_{p}^{2}$ = 0.6. Post hoc comparisons with Bonferroni correction showed that error rates in the standard condition (*M* = 0.12; *SD* = 0.07) were significantly higher than both the repeated condition (*M* = 0.08; *SD* = 0.06), *t*(33) =  − 4.08, *p* < 0.001, *d* = − 0.69, and the distractor absent condition (*M* = 0.06; *SD* = 0.04), *t*(33) =  − 5.22, *p* < 0.001, *d* = − 0.89. No significant difference was found between error rates in the distractor-absent condition and the repeated condition, *t*(33) =  − 1.80, *p* = 0.24 (see Fig. 2).

The results clearly showed that the attentional grabbing power of a color singleton was completely abolished when the singleton iteratively appeared at a fixed location, even when no competing target was present during such repeated presentations. Major models of visual saliency computation assume that attention is shifted to locations where local feature contrast are detected in the scene (e.g., Itti & Koch, 2001; Luck et al., 2021); hence, it can reasonably be assumed that at least initially the singleton captured attention also in the repeated condition. However, because such an element was totally irrelevant, a rejection process was initiated during the singleton repetitions to prevent the attention being oriented toward the salient element even if the target was absent. Given that the location of the singleton was fixed, the strong capture attenuation observed in the repeated condition may have arisen from a suppressive signal strategically or implicitly applied to the singleton location and/or color before the last display. Alternatively, as suggested by the habituation hypothesis (Sokolov et al., 2002), the singleton rejection could be controlled by an “expectation-based” mechanism, namely by the fact that in the repeated condition participants efficiently learned to anticipate the singleton occurrence in the last display, which does not require the concomitant presence of a target. Whatever the mechanism at play, the results indicates that the rejection is much more robust and rapid when the repetitions occur within the same trial (repeated condition) than across trials (standard condition), possibly indicating two rejection mechanisms operating on different time scales (also see Pascucci & Turatto, 2015).

**Fig. 3:**
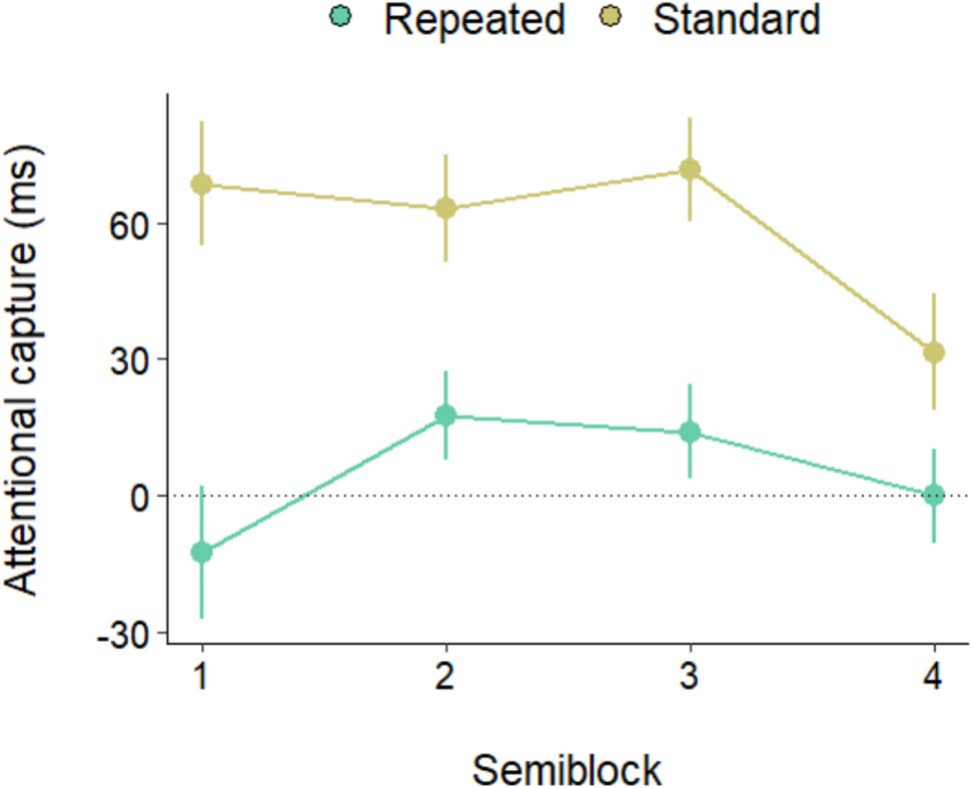
Attentional capture as a function of distractor condition and semiblock. Error bars denote the standard error of the mean

We therefore analyzed the time-course of capture attenuation in the repeated and standard conditions. The amount of capture in the two conditions was computed as the RT difference with the absent condition (RT present minus RT absent), as a function of time (we divided the data from the two blocks into four semiblocks to achieve a more fine-grained analysis). As is evident from Fig. 3, while in the repeated condition capture was already abolished in the first semiblock, in the standard condition the singleton continued to capture attention across semiblocks, although capture diminished in the last one. This pattern was confirmed by an ANOVA on the RT differences with semiblock and condition as factors, which showed a main effect of condition, *F*(1, 33) = 50.35, *p* < 0.001, ${\eta }_{p}^{2}$ = 0.60, and a close to significance Condition × Semiblock interaction,* F*(2.39, 78.91) = 2.84, *p* = 0.055, ${\eta }_{p}^{2}$ = 0.07. In the standard condition capture was significant in all blocks (all *p* values < 0.01), but it was smaller in Block 4 than in Block 1, *t*(33) = 2.01, *p* = 0.02. By contrast, in the repeated condition capture was already absent from the first block (all *p* values > 0.05). The super rapid rejection achieved because of the within-trial singleton repetitions is attested by the fact that in the repeated condition, capture was already completely abolished after 10 trials, *t*(33) = 0.46, *p* = 0.64, during which participants were exposed to just 40 color singletons; capture was instead still robust in the last semiblock of the standard condition, *t*(33) = 8.37, *p* ≤ 0.001, *d* = 1.4—namely, after that participants were exposed to 90 color singletons.

This pattern of results indicates that in the repeated conditions the singleton rejection was probably controlled by a faster (within trial) mechanism compared with the one operating (across trials) in the standard condition.

## Experiment 2

The results of Experiment 1 unequivocally showed that a color singleton distractor no longer captured attention when, before competing with the target for attention in the last display, the singleton was iteratively presented in the same location.

The advantage of the repeated condition might have emerged because participants possibly used the singleton repetitions before the last display to anticipate the distractor location, thus favoring a location-based rejection (e.g., Ferrante et al., 2018; Gaspelin & Luck, 2018; Sauter et al., 2019; Turatto & Valsecchi, 2021; Wang & Theeuwes, 2018), so that by the last display, when the competition with the target took place, the singleton no longer captured attention. This possibility was clearly precluded in the standard condition, because the first three displays were identical to the distractor-absent condition, and the singleton appeared only with the target in the last display.

Although color-singleton distractor rejection mechanisms can clearly operate based on the distractor spatial coordinates, rejection can also occur based on the distractor color statistics, irrespective of its spatial location (Stilwell et al., 2019). Hence, to address whether the advantage of the repeated condition observed in Experiment 1 persists also when any spatial based mechanisms is precluded, in the repeated condition of the present experiment, the color-singleton randomly changed its location during the four displays, with the constraint that, in each trial, it never appeared twice in the same location. Likewise, the singleton location in the standard condition also varied unpredictably from trial to trial. Hence, the location of the singleton in the last display, when it directly competed for attention with the target, was equally unpredictable in the repeated and standard conditions.

### Method

#### Participants

Thirty-four participants (15 female; 19 male; mean age 28,29) were recruited following the same procedure of Experiment 1. Four participants were excluded because they failed to reach the accuracy threshold and were replaced with new participants.

#### Stimuli and procedure

**Fig. 4:**
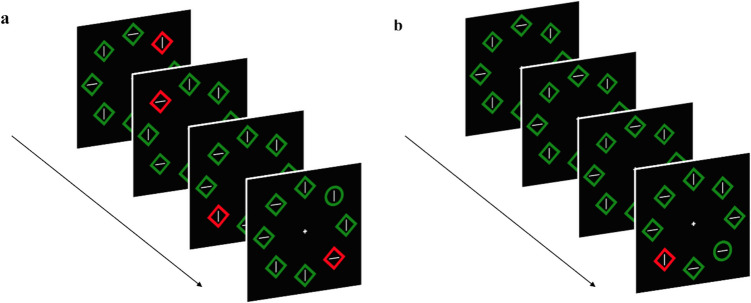
Schematic examples of a trial for the distractor-present experimental conditions. In the repeated condition (**a**), the color singleton appeared in a different position in each of the four displays. In the standard condition (**b**), the singleton appeared in a random location in the last display. The distractor-absent condition is not shown in the figure but was the same as in Experiment 1. (Color figure online)

Everything was the same as in Experiment 1, except that in the standard condition the distractor could randomly appear in one of the eight locations. In the repeated condition, the distractor position was different within each display, with the constraint that it could not appear twice on the same position in the same trial (see Fig. 4). Trials of each condition always appeared randomly intermixed.

### Results and discussion

**Fig. 5:**
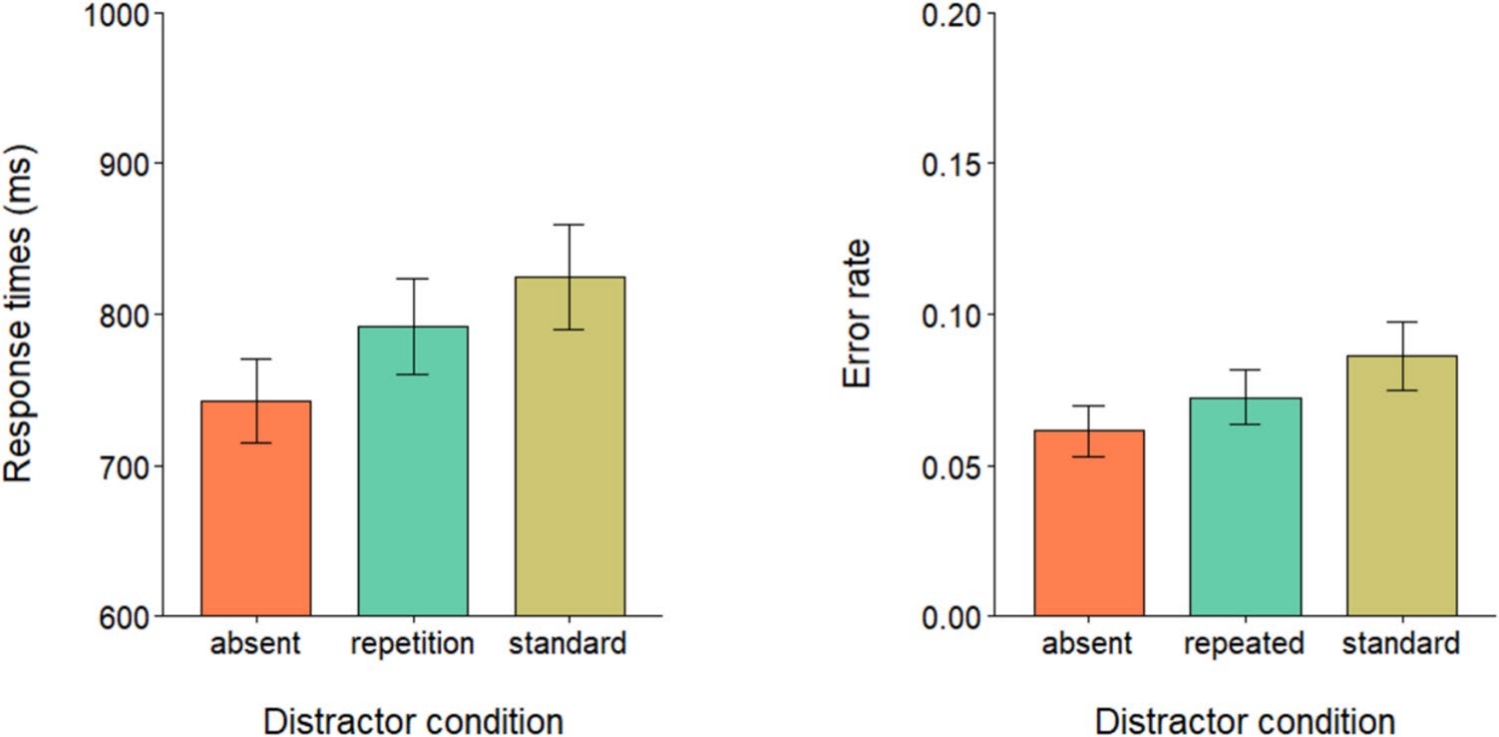
Mean RTs (left panel) and error rate proportions (right panel) in the three distractor conditions. Error bars denote the standard error of the mean

Correct RTs (mean accuracy = 92.77%) after outliers removal (< 1%) were entered into a repeated-measures ANOVA with the same factor levels as before: distractor absent, standard, and repeated. The results (see Fig. 5) revealed a significant main effect of distractor condition, *F*(2, 66) = 42.18, *p* ≤ 0.001, ${\eta }_{p}^{2}$ = 0.56. Post hoc comparisons with Bonferroni correction showed that in the standard condition (*M* = 824 ms; *SD* = 203 ms) participant were, as in Experiment 1, significantly slower than in the repeated condition (*M* = 791 ms; *SD* = 187 ms), *t*(33) =  − 5.25, *p* < 0.001, *d* = − 0.74, and in the distractor-absent condition (*M* = 742 ms; *SD* = 162 ms), *t*(33) =  − 8.31, *p* < 0.001, *d* = − 1.43. However, unlike in Experiment 1, in the repeated condition, participants were significantly slower than in the distractor-absent condition, *t*(33) =  − 4.35, *p* < 0.001, *d* = − 0.90. An ANOVA on error rates showed a main effect of distractor condition, *F*(2, 66) = 4.05, *p* = 0.02, ${\eta }_{p}^{2}$ = 0.10. Post hoc tests with Bonferroni correction showed that error rates were lower in the distractor-absent condition (*M* = 0.06; *SD* = 0.04) compared with the standard condition (*M* = 0.07; *SD* = 0.05), *t*(33) =  − 2.77, *p* = 0.01, *d* = − 0.47. No significant differences were found between the distractor absent and the repeated condition (*M* = 0.08; *SD* = 0.06), *t*(33) =  − 1.17, *p* = 0.74, and between the repeated and the standard condition, *t*(33) =  − 1.78, *p* = 0.25.

**Fig. 6:**
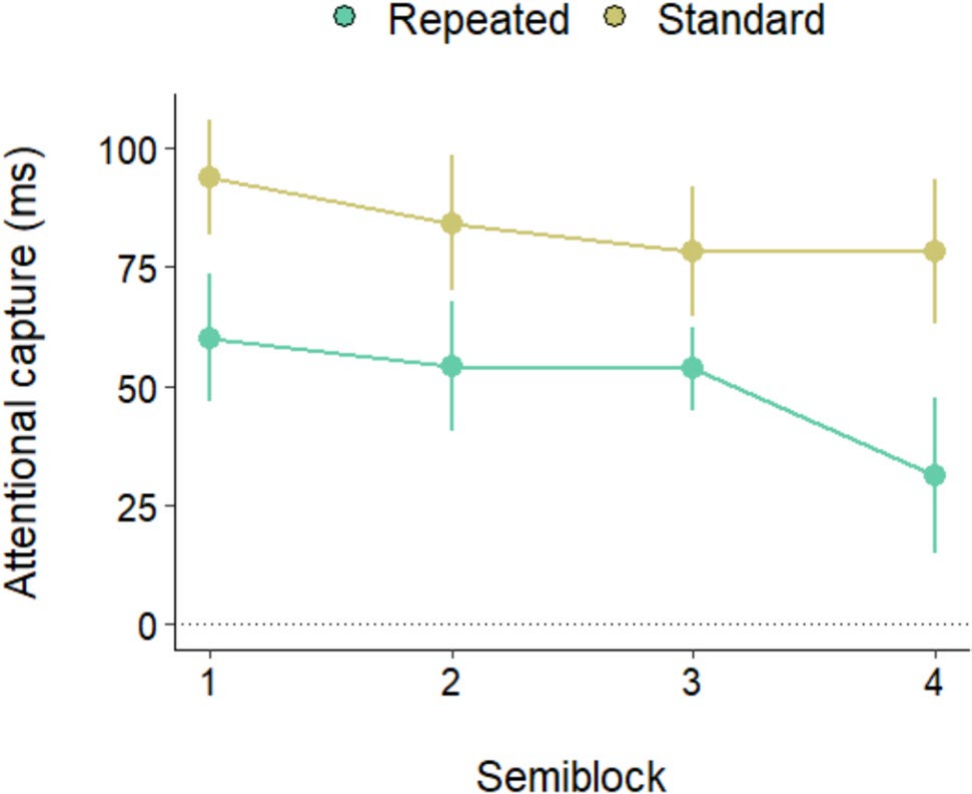
Attentional capture as a function of distractor condition and semiblock. Error bars denote the standard error of the mean

We also performed the same time-course analysis as in Experiment 1, in which attentional captures for the two distractor-present conditions across four semiblocks were entered into an ANOVA, which showed a significant main effect of distractor condition, *F*(1, 33) = 20.38, *p* ≤ 0.001, ${\eta }_{p}^{2}$ = 0.38, but no effect of semiblock, and the Condition × Semiblock interaction was not significant (see Fig. 6). Despite not being completely abolished as in Experiment 1, capture in the repeated condition was already significantly weaker than in the standard condition already in the first semiblock, *t*(33) = 2.02, *p* = 0.02, *d* = 0.34.

Overall, the results were consistent with those of the first experiment and confirmed that the attentional capture elicited by an irrelevant singleton was weakened when the singleton was repeatedly detected in the scene, even in the absence of a competing target element. Additionally, attentional capture in the repeated condition was attenuated, compared with the standard condition, even if the singleton changed its location during the repetitions. This clearly indicates that the within-trial singleton rejection mechanism can also operate independently from the singleton spatial coordinates. However, whereas in Experiment 1 the attentional capture was totally abolished in the repeated condition, in Experiment 2 capture was still present in this condition compared with the distractor-absent condition, which suggests that the rejection mechanism is more efficient when it can take advantage of spatial regularities.

**Fig. 7:**
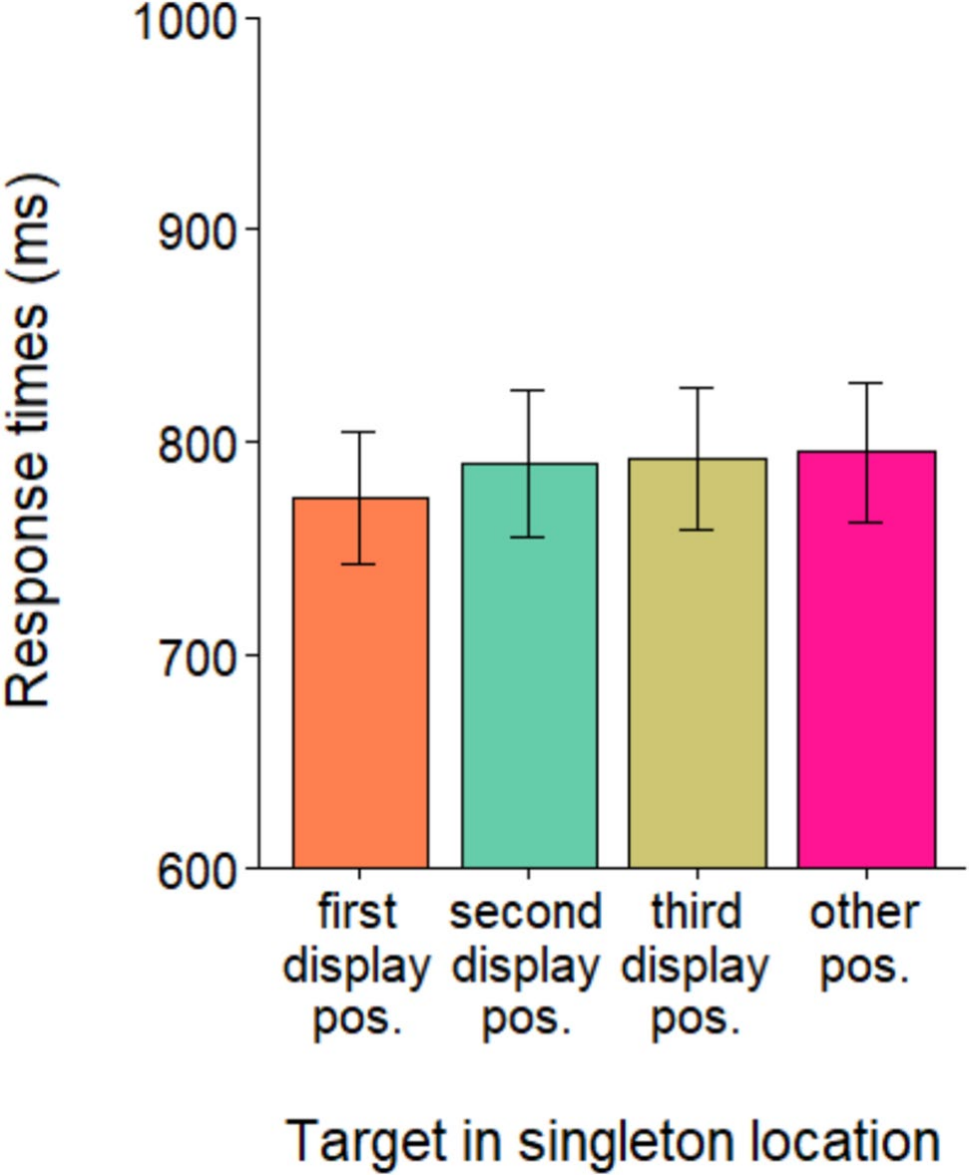
Mean RTs as a function of whether the target appeared in each of the locations where the singleton appeared in the first three displays or in one of the remaining locations. Error bars denote the standard error of the mean

In a further analysis of the repeated condition, we compared RTs when the target appeared in a position previously occupied by the singleton during the preceding three displays within the same trial, to RTs when it appeared in one of the remaining positions not occupied by the singleton in that trial (see Fig. 7). The reasoning was that if the weaker capture observed in the repeated condition was due to a suppressive mechanism applied to the singleton, an inhibitory trace would remain at the locations where the distractor appeared in first three displays. Consequently, if the target in the fourth display appeared in one of those positions, an impairment in its processing (reflected in slower RTs) should be observable. An ANOVA with location as factor comparing RTs when the target appeared in the location occupied by the singleton in the first, second or third display respectively, or in one of the remaining locations showed no effect of location, *F*(3, 99) = 1.0, *p* = 0.37.

## Experiment 3

So far, the results of Experiments 1 and 2 provided clear evidence that the rejection of a salient but irrelevant color singleton can occur even if this is not in direct competition with a shape singleton target for attention, as it typically does in the classic additional-singleton paradigm. This indicates that any rejection mechanism involved in reducing the attentional capture elicited by the color singleton is not necessarily engaged because the brain detects that attention has erroneously selected this salient element instead of the concurrently present target.

What remains unclear, however, is why rejection was much more efficient in the repeated condition compared with the standard condition. Different explanations are possible. For example, if on the one hand in Experiment 1 the location and color of the singleton were fixed in the repeated condition, the results of Experiment 2, where only the color was fixed, clearly show that the singleton rejection can take place even when the role of location is excluded. However, the fact that the singleton color was constant during the repetitions might have acted as a cue for the distractor in the last display, thus allowing to strategically anticipate the suppression. Similarly, the advantage of the repeated condition could result from a color-based suppression that in the repeated condition started with the first singleton appearance in the first display, and that may have accumulated through the within-trial repetitions, creating a sort of priming of suppression, which potentiated the singleton rejection.

Alternatively, an intriguing possibility is that capture attenuation in the repeated condition did not rely on suppressive mechanism but was rather controlled by a surprise- or expectation-based mechanism. In other words, capture was attenuated because through repetitions the brain might have rapidly generated an (even implicit) expectation about the upcoming appearance of an irrelevant color-singleton element (in a fixed or random location) in the last display, which would have made such singleton less unexpected or surprising and therefore less capable of attracting attention when it appeared with the target (Itti & Baldi, 2009; Sokolov, 1963). Because the first three displays of the standard condition were indistinguishable from those of the distractor-absent condition, the occurrence of the singleton in the last display of the standard condition could not be anticipated or expected, and its appearance captured attention.

To disentangle these alternative accounts, in the present experiment, the color of the singleton in the last display was made unpredictable. Specifically, in the repeated condition, the singleton appeared in a given color (which was fixed within-participants but changed across participants) in the first three displays (e.g., blue) and then randomly changed in the last display (e.g., to red or green). In the standard condition, the singleton color was randomly chosen between the two colors that could appear in the last display of the repeated condition (red or green in this example). Under these conditions, any advantage of the repeated condition over the standard one, if any, cannot be attributed to color-based suppression, color priming, or color cueing, since the color of the singleton in the last display was totally unpredictable. Conversely, if the singleton rejection is achieved by means of a prediction- or expectation-based mechanism, then we should still observe capture attenuation in the repeated condition. This is because during the within-trial repetitions participants may learn to expect a singleton in the last display, irrespective of its specific color, thus making it less surprising and therefore capable to attract attention.

### Method

#### Participants

Thirty-four participants (13 women; 21 men; mean age = 31.6 years) were recruited following the same procedure of Experiment 1. One participant was excluded because they failed to reach the accuracy threshold and was replaced with a new participant.

#### Stimuli and procedure

**Fig. 8:**
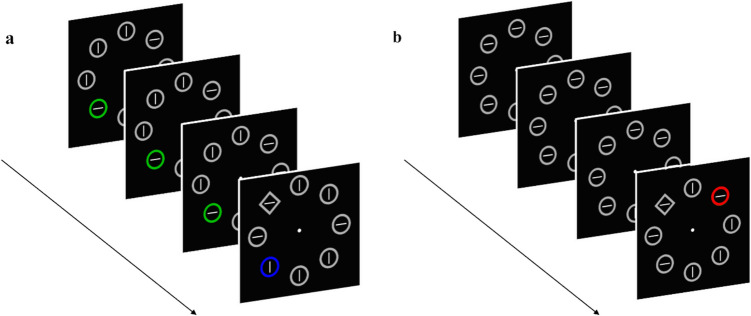
Schematic examples of a trial for the distractor-present experimental conditions. In the repeated condition (**a**), the color singleton appeared in a fixed position in each display within the same trial and had a fixed color (that remained fixed for the same participant but changed randomly across participants) for the first three displays (here, green), while the color of the distractor in the last display was chosen randomly between two other colors (here, red or blue). In the standard condition (**b**), the singleton color was chosen randomly between the same two colors of the final display of the repeated condition (here, red or blue). The distractor-absent condition (not shown in the figure) was the same as in Experiment 1, except that the non-target elements of the array were gray. (Color figure online)

Everything was the same as in Experiment 1, except for the following differences. The color of the array of figures was grey. In the repeated condition, the color of the distractor stayed the same in the first three displays (it was selected between red, green, and blue and remained fixed for each participant but changed randomly across participants). In the last display it changed randomly into one of the two remaining colors (e.g., red or blue, assuming in the first three displays it was green). In each trial of the standard condition, the singleton distractor color could be one of the two colors in the last display of the repeated condition, selected randomly (red or blue in this example; see Fig. 8). As in the previous two experiments, trials of each condition appeared randomly intermixed.

## Results and discussion

**Fig. 9:**
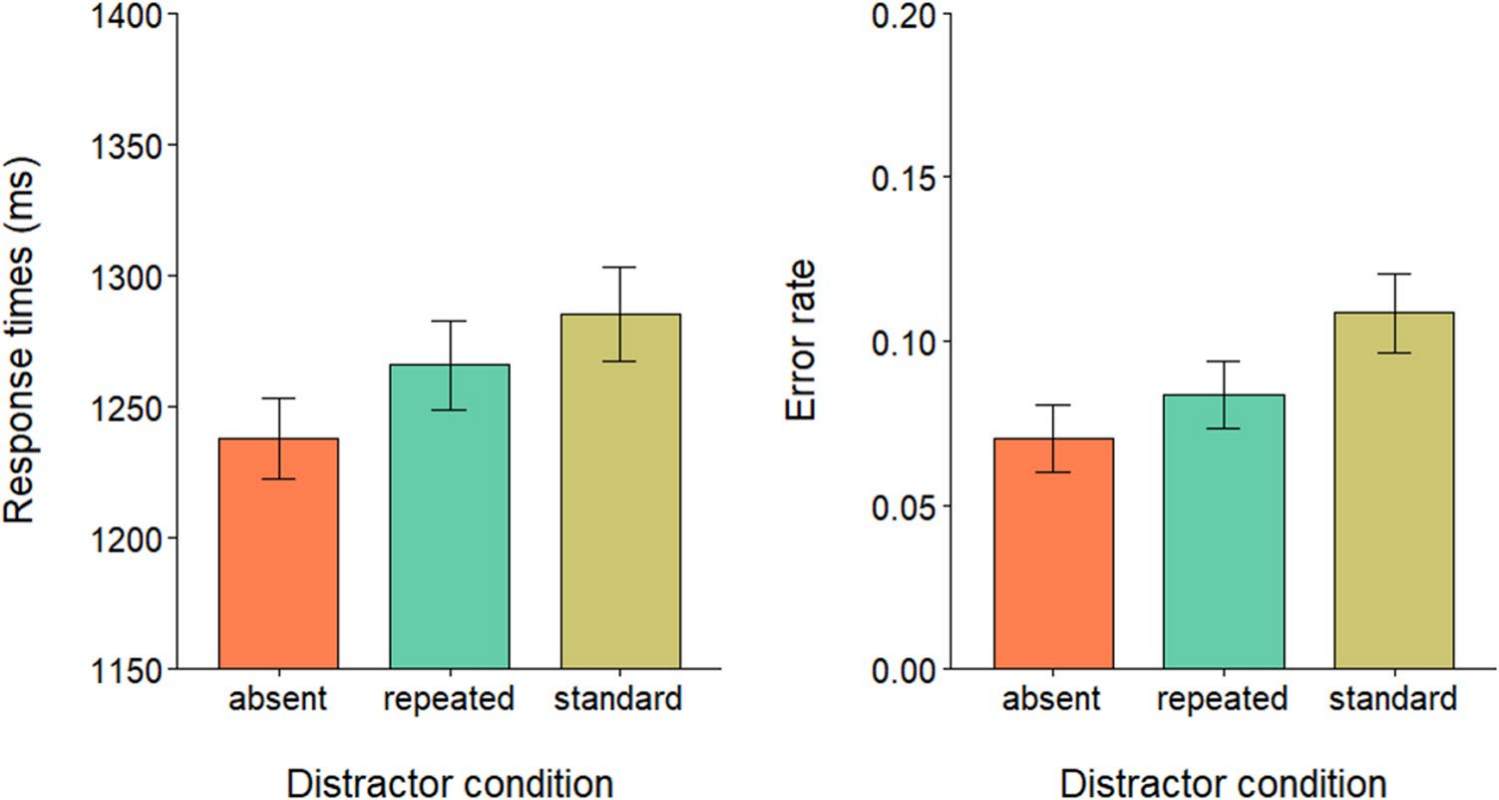
Mean RTs (left panel) and error rate proportions (right panel) in the three distractor conditions. Error bars denote the standard error of the mean

Correct RTs (mean accuracy = 90%) after outliers removal (1%) were entered into a repeated-measures ANOVA with the three conditions of distractor absent, repeated, and standard as factor levels. The results (see Fig. 9) revealed a significant main effect of distractor condition, *F*(2, 66) = 31.59, *p* ≤ 0.001, ${\eta }_{p}^{2}$ = 0.49. Post hoc comparisons with Bonferroni correction showed that in the standard condition (*M* = 1,285 ms; *SD* = 103 ms) participant were, as in Experiments 1 and 2, significantly slower than in the repeated condition (*M* = 1,265 ms; *SD* = 100 ms), *t*(33) =  − 3.31, *p* = 0.007, *d* = − 0.5, and in the distractor-absent condition (*M* = 1,237 ms; *SD* = 88 ms), *t*(33) =  − 7.51, *p* < 0.001, *d* = − 1.3. RTs in the repeated condition were significantly higher than those in the distractor-absent condition, *t*(33) =  − 4.83, *p* < 0.001, *d* = − 0.8. An ANOVA on error rates showed a significant main effect of distractor condition, *F*(2, 66) = 11.84, *p* ≤ 0.001, ${\eta }_{p}^{2}$ = 0.25. Post hoc comparisons with Bonferroni correction showed that error rates were higher in the standard condition compared with both the distractor-absent, *t*(33) =  − 5.31, *p* < 0.001, *d* = − 0.91, and the repeated condition, *t*(33) =  − 2.77, *p* = 0.02, *d* = − 0.47. There was no difference between the repeated and the distractor-absent condition, *t*(33) =  − 1.66, *p* = 0.31.

It should be noted that in Experiment 3, by making the distractor color unpredictable also in the repeated condition, we ensured that the comparison between that and the standard condition was more balanced. Indeed, while in the repeated condition of Experiments 1 and 2 the distractor color remained consistent during the within-trial repetitions, the distractor color changed randomly from trial to trial in the standard condition. However, even once this potential confounding factor was ruled out, we replicated the same finding of the previous experiments.

**Fig. 10:**
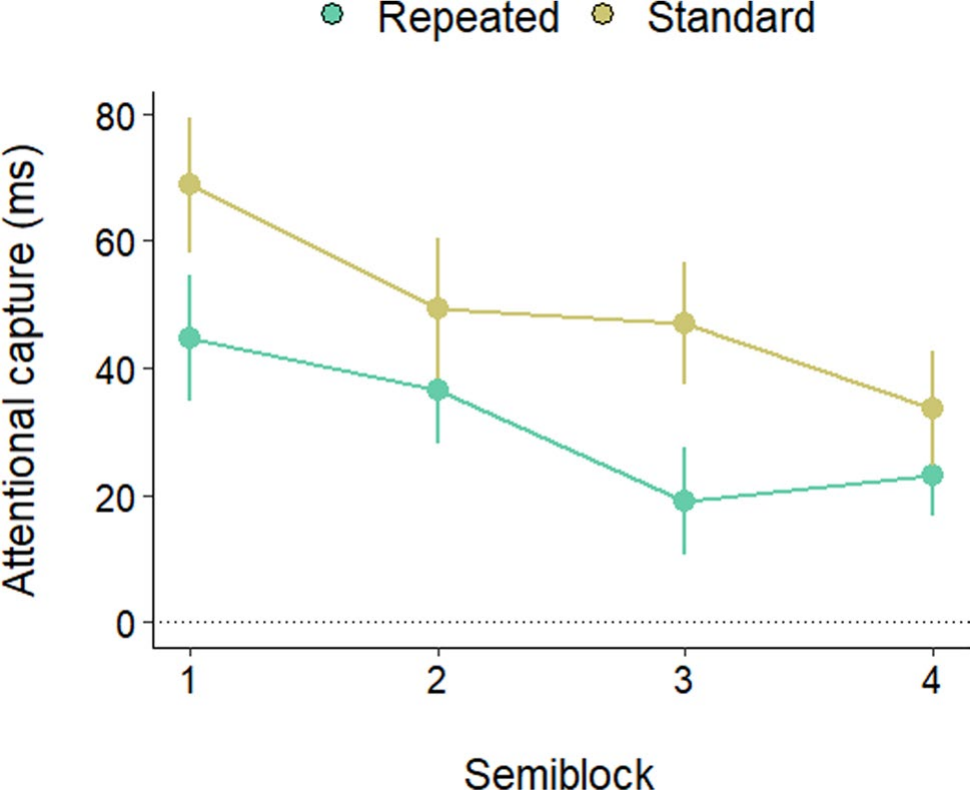
Attentional capture as a function of distractor condition and semiblock. Error bars denote the standard error of the mean

We then performed the same time-course analysis of capture as in Experiments 1 and 2. An ANOVA with condition (repeated vs. standard) and semiblock as factors showed a significant main effect of both condition, *F*(1, 33) = 9.85, *p* = 0.004, ${\eta }_{p}^{2}$ = 0.23, and semiblock, *F*(2.62, 86.41) = 3.70, *p* = 0.01, ${\eta }_{p}^{2}$ = 0.10, while the Condition × Semiblock interaction was not significant (see Fig. 10). The results thus showed that capture diminished with practice in both conditions, but it is worth noting that, as shown in Experiments 1 and 2, capture was already significantly smaller in the repeated condition compared with the standard one already from the first semiblock, as shown by a one-tailed *t* test, *t*(33) = 2.07, *p* = 0.02, *d* = 0.35, confirming the faster rejection in the former case despite the color in the last display was unpredictable.

## General discussion

This study aimed to test whether the rejection of a feature-singleton distractor is driven by the misallocation of attention to this salient element relative to a concurrently present target (e.g., Allenmark et al., 2022; Luck et al., 2021), as typically occurs in the widely used additional-singleton paradigm (e.g., Theeuwes, 1992). In this regard, the results of the three experiments we have conducted consistently showed that the capture elicited by a color singleton, either because of its salience or because participants were engaged in a singleton search (Bacon & Egeth, 1994) or both, can be attenuated, and even entirely abolished when the singleton was encountered iteratively within the same trial, with no target present in the display.

Some previous studies have already demonstrated distractor rejection in the absence of a competing target. For example, when participants were passively exposed to two blocks of trials featuring salient peripheral onsets without a target, onset capture was already significantly attenuated when the target was subsequently introduced in later blocks, consistent with the habituation of capture (Turatto, 2023; Turatto et al., 2018a, 2018b). Regarding feature-singleton distractors, rejection in the absence of a competing target was recently reported in a study by Tsai et al. (2023), where participants were asked to detect the presence or absence of a shape-singleton target. The results indicated that a singleton distractor captured attention when it was defined within the same dimension as the target (shape), but not when defined in a different dimension (color). These findings, compatible with the dimension-weighting account (Liesefeld & Müller, 2019), attested a complete rejection when the distractor was in a different dimension as the target, even when the latter was absent. Our study, however, differs from the few previous studies reporting evidence of distractor rejection in the absence of a competing target because here the stronger capture attenuation was achieved through the within-trial singleton repetitions rather than its across-trial repetitions. This, together with the different time-course of rejection in the two cases, might suggest, as discussed below, the existence of rejection mechanisms that could operate on different time scales, within trial and across trials.

With respect to the possibility that a singleton rejection can take place in the absence of a competing target it could be argued that such rejection is not necessarily triggered by a misallocation of attention relative to the target in the display, but relative to the ‘task set’, which involves finding the target in the scene. This task set would remain active throughout the experiment, including during the first three displays in the repeated condition of the present experiments, even when no target was shown. However, if an “erroneous” selection is defined not in relation to a concurrent competing target but rather with respect to a task set, then this implies that the singleton is rejected simply because it is irrelevant, as postulated by habituation theories long ago. These assume that an irrelevant repetitive stimulus (in relation to a current goal) is progressively ignored, with the corresponding orienting response being attenuated (Sokolov, 1963). The possibility that the rejection of a salient element does not necessarily depend on the presence of a target, or any related task set, is supported by the study of Turatto et al., (2018a, 2018b), in which participants learned to filter an irrelevant onset distractor during passive viewing (even when no fixation was required). In Turatto et al.’s study the potential presence of a “to-be-selected” target was never mentioned, and therefore no target-related task set was active. Although participants were not expecting the occurrence of a target to discriminate, simply allowing them to experience the repetitive occurrence of an irrelevant salient element was sufficient for them to learn to disregard this event.

Regarding the specific distractor rejection mechanism responsible for the strong capture attenuation emerged in the repeated condition of the present study, it is worth considering different possibilities. Among the various mechanisms that have been proposed for reducing the negative impact of a distractor, the most prominent one assumes that the distractor salience is attenuated via suppressive signals applied at the saliency map level, or at lower dimension-based levels, in the saliency-computation architecture (e.g., Chelazzi et al., 2019; Gaspelin & Luck, 2018; Geng, 2014; Liesefeld & Müller, 2019; van Moorselaar & Slagter, 2020). According to this view, the amount of suppression would be proportional to the rate of distractor occurrence, across trials, in a given location (e.g., Wang & Theeuwes, 2018) or color (e.g., Stilwell et al., 2019).

A suppressive mechanism could therefore underlie also the reduced capture observed in the repeated condition of our experiments. The results of both Experiments 1 and 2 are in principle compatible with a color-based suppression hypothesis, since the singleton color remained constant during the within-trial repetitions. Suppression was likely stronger in Experiment 1 because the distractor appeared in a fixed location, a regularity that can be exploited to enhance suppression further. However, Experiment 2 demonstrated that a singleton capture attenuation was also possible when the salient, irrelevant element appeared in different locations during the within-trial repetitions. Furthermore, since the singleton color remained constant, it may have served as an implicit cue, allowing for a preemptive suppressive response before the final display, where the singleton competed with the target for attention. This likely enabled a more efficient rejection compared with the standard condition.

Experiment 3 was therefore designed to examine the roles of cueing and suppression in capture attenuation when the color singleton repeated within the same trial. Because the color of the singleton in the final display was unpredictable, in both the repeated and standard conditions, this excludes the possibility that the advantage observed in the repeated condition was due to color-based suppression or a cueing effect based on color repetition during the first three displays. Additional observations also suggest that reduced capture in the repeated condition is not explained by a suppressive signal applied to the singleton location. First, in Experiment 1 we found no evidence of target processing impairment when, in distractor-absent trials, the target appeared at the repeated singleton location compared with other locations. Second, in the repeated condition of Experiment 2, no target processing impairment occurred when the target appeared at any of the positions previously occupied by the singleton during the first three displays. By contrast, previous studies using the classic additional-singleton paradigm have consistently reported a delay in target processing when it appears, in distractor-absent trials, at the position more frequently occupied by the color distractor. This delay is typically taken as proof that the weaker capture observed at this location, compared with less frequently occupied positions, is due to distractor suppression (e.g., Ferrante et al., 2018; van Moorselaar & Theeuwes, 2024; Wang & Theeuwes, 2018; Zhang et al., 2019).

The random color change of the singleton in the final display excludes that the observed advantage in the repeated condition can be explained by the distractor preview effect (DPE; Ariga & Kawahara, 2004; Wan & Lleras, 2010) as well. The DPE refers to attentional inhibition directed at features previewed in a target-absent display during singleton feature searches. For example, if participants encounter a display without a singleton (e.g., all items are red), the feature associated with the distractors (red) becomes inhibited. In subsequent trials, this results in slower selection of a target with that feature, while distractors sharing such feature are more easily ignored.

Drawing a direct parallel between the DPE and the within-trial repetitions in our study is problematic due to procedural differences, but it is worth considering that the mechanism underlying DPE relies on attention being biased away from a previously rejected feature. Thus, selection of a target with such a feature is delayed, whereas focusing on a target surrounded by distractors defined by this feature is facilitated. There are two main ways the DPE could potentially influence our findings. First, the target in the final display might have been avoided because it shared a previously rejected feature. However, this is unlikely since the target appeared as singleton shape only in the last display (e.g., a diamond among circles), and the other items’ shapes remained constant and uniform throughout the trial. Consequently, the shape of the target could not have been recently rejected, as it was never previewed. One might argue that the target color could have induced this effect: items in target-absent displays were the same color of the target (e.g., green), so this previewed color was later rejected. This is implausible for two reasons: First, the previewed color was also shared with other display items, so any effect should have concerned every stimulus in the display. Second, color was not a relevant feature for the search task, and task relevance is a prerequisite for DPE (Levinthal & Lleras, 2008).

A second possibility involves distractor previewing. However, this explanation seems also inadequate. By definition, DPE arises from previewing displays without a singleton, as demonstrated by Goolsby et al. (2005), who found that the DPE occurs only when uniformly colored displays are previewed. In our Experiments 1 and 2, however, the distractor color was previewed only as a color singleton, not as part of a uniform array. Moreover, as already noted, the DPE is effective only when the inhibited feature is task relevant. Given that our task relied on shape rather than color, any color preview should not have generated a DPE. If any effect was induced, it would have been linked to the previewed search-relevant feature (e.g., circle), but note that this occurred in all our experimental conditions (i.e., repeated, single, absent). Finally, even if one were to assume that color might have caused a DPE-like effect despite its irrelevance to the task, this explanation is ruled out by Experiment 3, where the distractor color was never previewed due to the color change in the final display of the repeated condition. This interpretation suggests that the observed effects likely arise from other attentional mechanisms.

If distractor suppression, cueing, or DPE seem insufficient to explain the stronger capture attenuation observed in the repeated condition, what alternative mechanism could account for this finding? We believe that the overall pattern of results from the three experiments supports the possibility that, at the within-trial level, the attenuation of capture elicited by the singleton was controlled by an “expectation-based” mechanism that anticipated the presence of an irrelevant salient element in the final display. This view aligns with the habituation account proposed by Sokolov in his *stimulus-model comparator* theory (Sokolov, 1963), according to which the more an irrelevant stimulus is repeated (in this case, the color singleton), the more it becomes expected, and thus the more effectively it can be ignored, meaning that attention is less likely to be drawn to a predictable salient event. Exogenous attention is indeed preferentially directed, at least initially, toward unexpected stimuli, whereas the orienting response (or orienting reflex) weakens for anticipated inputs (Sokolov, 1963; Sokolov et al., 2002). Several studies have provided evidence that an habituation mechanism based on distractor expectation can provide a straightforward explanation for the capture attenuation observed with onset distractors when these are repeated across trials, but evidence also exists that Sokolov-like habituation mechanisms could also play a role in case of feature singleton distractors (e.g., De Tommaso & Turatto, 2019; Folk & Remington, 2015; Kelley & Yantis, 2009; Pascucci & Turatto, 2015; Turatto, 2023; Turatto & De Tommaso, 2023; Turatto & Pascucci, 2016; Turatto et al., 2018a, 2018b; Valsecchi & Turatto, 2022; Won, 2021; Won & Geng, 2020). With respect to Sokolov’s idea, which suggests that the more an irrelevant input is expected, the more the resultant orienting response is inhibited, we propose that, at least in the case of within-trial repetitions, such suppression might not be strictly necessary to modulate capture. Instead, the strength of the capture response elicited by the distractor would be determined by how much the salient event is expected or, more precisely, by how surprising it is. The role of distractor expectation in the distractor rejection mechanism aligns with the idea that attention is attracted to unexpected or surprising events (Itti & Baldi, 2009). In other words, attention would be attracted to the singleton in proportion to the “singleton prediction error,” which was progressively attenuated during the within-trial repetitions in the repeated condition. Crucially, the singleton prediction error was maximal in the fourth display of the standard condition because the first three repetitions of distractor-absent and standard trials were indistinguishable. As a result, the appearance of the singleton in the fourth display of the latter type of trial was somewhat surprising.

The hypothesis that the within-trial singleton rejection was governed by an “expectation-based” mechanism aligns with the finding that capture was significantly more attenuated in Experiment 1 compared with Experiments 2 and 3. In Experiment 1, the singleton consistently appeared in the same color and location, thereby reducing the level of surprise associated with its appearance. In contrast, the singleton location was unpredictable in Experiment 2, and its color in the last display was unpredictable in Experiment 3. These spatial and color uncertainties likely increased the distractor-prediction error, leading to less effective distractor rejection.

Hence, while previous studies, in which the distractor is typically repeated across trials, have provided evidence consistent with the role of a suppressive signal applied to the distractor based on its statistics (e.g., Ferrante et al., 2018; van Moorselaar & Theeuwes, 2024; Wang & Theeuwes, 2018; Zhang et al., 2019), the present findings in the present study we did not find evidence of suppression being involved in distractor rejection, which seems to be governed simply on the basis the surprise associated with the distractor presence within a restricted time window (i.e., within the trial).

That the rejection mechanism based on within-trial singleton repetition might differ from the one based on across-trial singleton repetitions is supported also by the time-course analysis of capture that we conducted. This revealed that capture attenuation was larger in the repeated condition compared with the standard condition from the first trials onward. For example, in the repeated condition of Experiment 1, capture was already eliminated (− 9 ms) after the first 10 trials of the first block, whereas it remained significant in the last semiblock of the standard condition. This rapid rejection emerged despite participants in the repeated condition being exposed to only 40 color singletons (four per trial), while participants in the last semiblock of the standard condition had been presented with 90 color singletons (one per 90 trials). Note that the faster rejection caused by the repeated condition was evident in each experiment, and that this result indicates that the within-trial repetitions did not merely accelerate the across-trials rejection mechanism or boost the effect of across-trial distractor presentations.

The possibility that in the repeated condition the singleton rejection was controlled by a specific mechanism different from the one operating across trials could also explain why in the repeated condition we found a strong capture attenuation, whereas De Tommaso and Turatto (2019) did not report any capture reduction when the singleton was repeated across trials, although it is conceivable that in both cases participants were engaged in a singleton search.

One may wonder whether the beneficial effect of repeating the singleton within the same trial reported here might be an extreme case of the intertrial sequence effects found in previous studies, where the distractor interference is reduced on a given distractor-present trial following another distractor-present trial compared with a distractor-absent trial, suggesting that rejection is modulated by trial history (e.g., Müller et al., 2009). At present, we cannot exclude this possibility, which needs to be specifically addressed in future studies, but in the attempt to provide a preliminary answer to this question we compared the amount of capture observed in the repeated condition with that found in the standard condition considering only, in the latter case, distractor-present trials that followed another distractor-present trial. The results showed that capture in the in the standard condition (*M* = 780 ms; *SD* = 148) was still stronger than in the repeated condition (*M* = 728 ms; *SD* = 129; *p* < 0.001), suggesting that the effects of within-trial singleton repetitions might have a different origin.

In sum, our study shows that distractor rejection does not require the concurrent presence of a competing target to be implemented; furthermore, the results point to the possible existence of within-trial and across-trials rejection mechanisms that operate on different time scales and through different processes, although we acknowledge that present this conclusion is still speculative. While previous findings indicate that rejection based on distractor statistics across trials is largely controlled by suppressive signals, rejection based on within-trial repetition may be not, being instead controlled by distractor expectation or prediction error.

## Data Availability

Data have been made publicly available via OSF and can be accessed online (https://osf.io/yzcfs/?view_only=b368a6ff44e14bd196c8caedc09140fa).

## Declarations

### Open practices statement

None of the experiments were preregistered. Data for all experiments have been made available and can be accessed online (https://osf.io/yzcfs/?view_only=b368a6ff44e14bd196c8caedc09140fa).
